# Transsphenoidal versus Transcranial Approach for Treatment of Tuberculum Sellae Meningiomas: A Systematic Review and Meta-analysis of Comparative Studies

**DOI:** 10.1038/s41598-019-41292-0

**Published:** 2019-03-19

**Authors:** Chengxian Yang, Yanghua Fan, Zhiwei Shen, Renzhi Wang, Xinjie Bao

**Affiliations:** Department of Neurosurgery, China Pituitary Disease Registry Center, Peking Union Medical College Hospital, Peking Union Medical College & Chinese Academy of Medical Sciences, Beijing, 100730 China

## Abstract

There is controversy regarding the surgical route selection for tuberculum sellae meningiomas (TSMs): the transsphenoidal (TS) or transcranial (TC) approach? We conducted a systematic review and meta-analysis to compare clinical outcomes and postoperative complications between two surgical approaches. Literature search was performed. Relevant articles were selected and evaluated. Data were extracted and analyzed. Eight articles comprising 550 patients met the inclusion criteria. Traditionally, the rates of gross total resection, tumor recurrence, visual improvement, and cerebrospinal fluid leakage were the most common outcomes of interest. We demonstrated that the TS approach was significantly associated with better visual outcomes but more frequent cerebrospinal fluid leakage, while the rates of tumor resection and recurrence showed no significant difference between groups. In addition to surgical results that were consistent with previous studies, we further evaluated the impact of approach selection on common postoperative complications, which were closely related to the recovery course and quality of life. We revealed that the risk of dysosmia was significantly higher in the TS group. There was no significant difference between groups regarding infection, intracranial hemorrhage, and endocrine disorders. Because of the relatively low evidence levels of included retrospective studies, it was difficult to reach a categorical conclusion about the optimal surgical approach for TSMs. Finally, we recommended that the TS approach was an alternative option in patients with smaller TSMs (<30 mm) and limited invasion of optic canals in experienced neurosurgical centers.

## Introduction

Tuberculum sellae meningiomas (TSMs) represent 5 to 10% of all intracranial meningiomas, invading the optic canals and displacing the optic nerves upward and laterally^1^. Therefore, despite of the relatively small proportion, such lesions are deeply concerned because of visual impairment in most cases^2^. Tumor resection and visual restoration are the two primary goals of the surgical treatment of TSMs. Traditionally, the transcranial approach (TC) has been the standard surgical route of removing TSMs and has achieved good outcomes^3–5^. In recent years, with the accumulation of endoscopic techniques and experiences, the transsphenoidal approach (TS) has been proposed for the resection of TSMs because of its minimal invasive nature^6–10^. Compared with TC, TS has some inherent advantages, such as minimized brain retraction, little optic apparatus manipulation, and direct removal of affected bone and dura. However, TS is more frequently associated with cerebrospinal fluid (CSF) leakage, which leads to a high risk of infection and may require a secondary repair. Nevertheless, with remarkable advances in skull base reconstruction, TS is considered as an important option for TSM resection^8,10–12^. Thus, there is a debate about the approach selection for TSMs^13–15^.

Although previous systematic reviews provided valuable conclusions in the approach selection for TSMs, each review had some limitations, of which the greatest one was the inability of calculation of overall odds ratio (OR) because of including non-comparative case series^16–20^. As the endoscopic technology matures, practitioners have reported more cases to directly compare the two approaches^15,21,22^. Herein, we performed a systematic review and meta-analysis of comparative studies regarding the approach selection for TSMs.

## Methods

### Search strategy

Our review is in accordance with the Preferred Reporting Items for Systematic Reviews and Meta-Analyses (PRISMA) Statement^23^. We performed a systematic review of literature using Pubmed and Embase from inception to June 21, 2018. The following search terms in various combinations were used: tuberculum sellae, meningioma, endoscopy, endoscopic, endonasal, minimal invasive, transsphenoidal, transcranial, and craniotomy. Only the English-language articles were included. Two independent researchers (C.Y. & Z.S.) performed the literature searches separately. If there was any discrepancy regarding the eligibility of an article, consensus was reached with the guidance of the senior authors (X.B. & R.W.).

### Inclusion criteria, data extraction, and quality assessment

**Figure 1:**
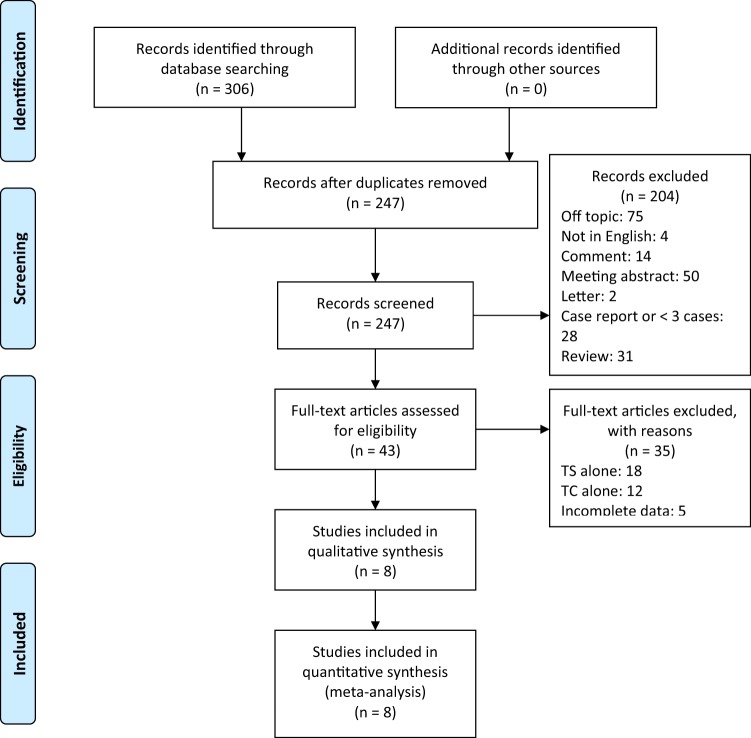
Flow diagram of search strategy.

The goals of the literature search were to find articles that met the following inclusion criteria: (1) described a comparative study including TSM patients treated by TS or TC approaches; (2) reported the number of patients and included at least three patients for each group; (3) reported the number of events for each group. Therefore, conference abstracts, non-comparative studies, and case reports were excluded. A flow chart of study selection process is shown in Fig. 1. Two authors (C.Y. & Z.S.) extracted relevant data from the selected studies independently and created a meta-analysis database with the following categories: (1) fundamental literature information including first author, publication year, and country; (2) characteristics of TSM patients including the number of total and female patients, age, tumor size, the number of patients with optic canal invasion and visual disturbance, and duration of follow-up; (3) data of clinical outcomes including the number of patients with gross total resection, improved visual outcomes, and recurrence for each group; (4) data of postoperative complications including the number of patients with CSF leakage, dysosmia, infection, intracranial hemorrhage, and endocrine disorders for each group. The 9-star Newcastle–Ottawa Scale (NOS) was used to assess the quality of eligible publications^24^. In this meta-analysis, studies with scores of 6 or more were considered as high-quality.

### Data analysis

We performed the statistical analyses of pooled data to compare the surgical outcomes and postoperative complications between TS and TC groups using Review Manager, version 5.3.5 (The Nordic Cochrane Centre, The Cochrane Collaboration, 2014). The overall OR was computed using the method of Mantel-Haenszel test. The random-effects model was performed. Study heterogeneity was determined using the Cochrane Q and I^2^ statistics. Heterogeneity was considered significant when the p value from Cochran Q was <0.1 or I^2^ > 50%. A subgroup analysis and a sensitivity analysis were used to find the main source of between-study heterogeneity. Publication bias was assessed by visually inspecting the funnel plots^25–27^.

## Results

### Literature search

Our search strategy initially identified 306 articles, of which 59 duplicated articles were excluded. After the initial screening of titles and abstracts, 204 articles were excluded because of unrelated subject matter, inappropriate article types and none-English languages. The remaining 43 articles were reviewed in full text and assessed for eligibility. Another 35 articles were excluded for insufficient data and non-comparative study designs. The selection process yielded 8 articles comprising 550 patients for inclusion^15,21,22,28–32^. Among the general population, 2 patients underwent both approaches, and the relevant data of TS and TC surgeries were separately analyzed in each group.

### Baseline data of included studies

The TS group comprised 220 patients, and the TC group comprised 332 patients. The number of female patients was available in 7 studies with an overall female proportion of 79.8%, indicating that women are predisposed to TSMs. The mean age in each group was reported in 6 studies, with age ranging from 23–82 years in the general patients. The detailed characteristics of included studies are presented in Table 1.

**Table 1:** Characteristics of included studies.

Authors & Year	Country	Years	No. of Ptx	TS	TC	Female	Age (mean ± SD)	Age (range)	No. of visual disturbance	Tumor size (p value)	Optic canal invasion (p value)	Follow-up (median)	Follow-up (range)	NOS scores
Song *et al*. 2018	Korea	2004–2015	84	44	40	72	TS: 53; TC: 54	24–76	78	TS: 25 ± 6 mm; TC: 26 ± 8 mm (p = 0.570)	TS: 34/44; TC: 32/40 (p = 0.832)	TS: 27; TC: 44	0–147	9
Magill *et al*. 2018	US	1997–2016	139	44	95	NA	NA	NA	121	NA	TS: 26/44; TC: 86/95 (p < 0.001; p = 0.177 regarding severity)	29; 46 (mean)	0–174	7
Kong *et al*. 2018	Korea	2010–2016	178	84	94	136	TS: 54 ± 14; TC: 54 ± 11	31–79	157	TS: 24 ± 7 mm; TC: 21 ± 8 mm (p > 0.05)	TS: 60/84; TC: 51/94 (p = 0.013)	28 (mean)	3–71	9
Linsler *et al*. 2017	Germany	2011–2016	22	6	16	17	TS: 66 ± 12; TC: 60 ± 12	46–82	8	TS: 2.1 ± 0.8 cm^3^; TC: 14.9 ± 8.2 cm^3^ (p < 0.05)	NA	18 ± 14 (mean ± SD)	3–60	8
Bowers *et al*. 2011	US	2002–2010	27	5	22	22	TS: 58 ± 17; TC: 53 ± 13	23–77	23	TS: 25 ± 7 mm; TC: 31 ± 13 mm (p = 0.945)	NA	NA	12–120	9
Fatemi *et al*. 2009	US	2000–2008	21	14	9	16	TS: 51 ± 15; TC: 49 ± 7	31–77	19	TS: 25 ± 8 mm; TC: 33 ± 10 mm (p = 0.008)	NA	TS: 28; TC: 14	TS: 6–65; TC: 3–28	8
Divitiis *et al*. 2008	Italy	1983–2006	51	7	44	41	NA	NA	51	TC: < 2 cm 6 cases; 2–4 cm 33 cases; > 4 cm 5 cases; TS: < 2 cm 2 cases; 2–4 cm 5 cases	TS: 1/7; TC: 2/44 (p = 0.364)	NA	TS: 0.75–20; TC: 9–252	8
Kitano *et al*. 2007	Japan	1994–2006	28	16	12	24	TS: 54 ± 10; TC: 61 ± 9	42–76	26	TC: 8.9 ± 9.4 mm^3^; TS: 7.5 ± 5.4 mm^3^ (p = 0.435)	NA	NA	TS: 3–96; TC: 108–156	9

Pts = patients; TS = transsphenoidal; TC = transcranial; NA = not available; NOS = Newcastle-Ottawa Scale; Unit of age = years; Unit of follow-up = months; Fatemi *et al*.: Two patients underwent both approaches; Kitano *et al*.: tumor volume = length × height × width/2; Linsler *et al*.: tumor volume = 3/4 × π × length × height × width.

#### Meta-analyses of clinical outcomes

**Figure 2:**
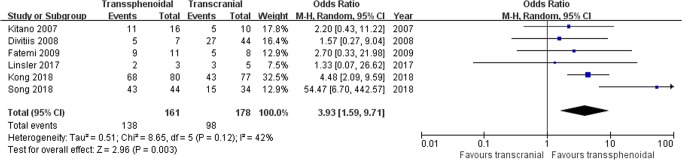
Forest plot of all studies with their respective OR and 95% CI, the number of events (visual improvement), and overall OR. OR = odds ratio; CI = confidence interval; M-H = Mantel-Haenszel.

Visual outcome: Six studies comprising 339 patients were included for the random-effects meta-analysis^15,21,28,29,31,32^. The rate of visual improvement in the TS group was 138/161 (85.7%), and it was 98/178 (55.1%) in the TC group. The meta-analysis of pooled data showed a significant benefit from the TS approach in the rate of improved visual function (OR 3.93, 95% CI 1.59–9.71; p = 0.003; Fig. 2). The I^2^ statistic of 42% indicated no significant heterogeneity among included studies.

**Figure 3:**
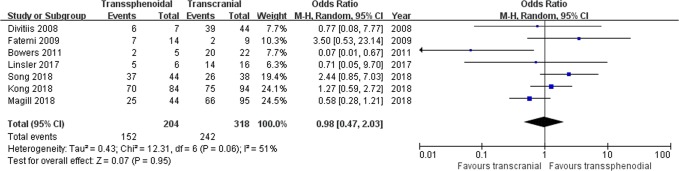
Forest plot of all studies with their respective OR and 95% CI, the number of events (gross total resection), and overall OR.

**Figure 4:**
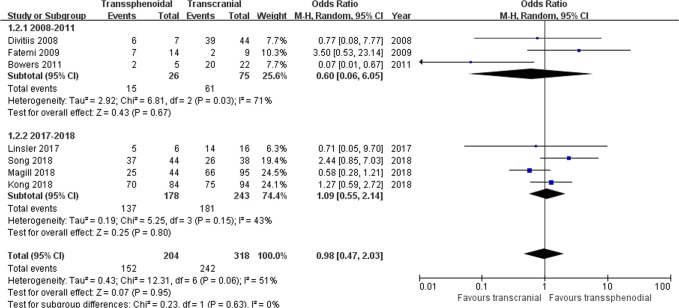
Subgroup analysis of gross total resection by the publication year.

Tumor resection: Seven studies comprising 522 patients were included for the random-effects meta-analysis^15,21,22,28–31^. The rate of gross total resection (GTR) in the TS group was 152/204 (74.5%), and it was 242/318 (76.1%) in the TC group. No significant difference was detected in the rate of GTR between the two groups (OR 0.98, 95% CI 0.47–2.03; p = 0.95; Fig. 3). The I^2^ statistic of 51% indicated significant heterogeneity among included studies. In the subgroup analysis, we divided the included studies into two groups according to the publication year range: group 1 (2008–2011) and group 2 (2017–2018). Significant between-study heterogeneity was detected in group 1 (p = 0.03, I^2^ = 71%), but not in group 2 (p = 0.15, I^2^ = 43%) (Fig. 4). The further sensitivity analysis in Table 2 revealed that the study by Bowers *et al*. was the main resource of heterogeneity. After removing the study by Bowers *et al*., there was still no significant difference in the rate of GTR (OR 1.16, 95% CI 0.66–2.07; p = 0.23), and then the I^2^ statistic of 27% indicated no significant heterogeneity among remaining studies.

**Table 2:** Sensitivity analysis comparison of TS and TC approaches regarding gross total resection.

Removed study	Overall effect of remaining studies (TS versus TC)		Study heterogeneity	
	OR (95% CI)	P value	I^2^	P value
Divitiis 2008	0.99 (0.44–2.21)	0.97	59%	0.03
Fatemi 2009	0.85 (0.39–1.81)	0.67	52%	0.06
Bowers 2011	1.16 (0.66–2.07)	0.23	27%	0.60
Linsler 2017	0.99 (0.44–2.20)	0.98	59%	0.03
Song 2018	0.79 (0.36–1.71)	0.55	44%	0.11
Kong 2018	0.87 (0.33–2.34)	0.79	57%	0.04
Magill 2018	1.14 (0.48–2.72)	0.77	46%	0.10

**Figure 5:**
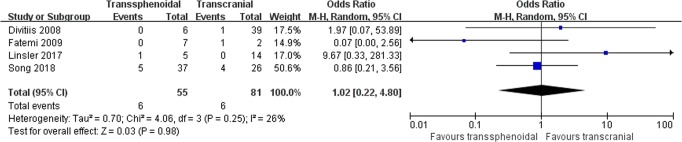
Forest plot of all studies with their respective OR and 95% CI, the number of events (tumor recurrence), and overall OR.

Recurrence: Four studies comprising 136 patients were included for the random-effects meta-analysis^15,21,29,31^. The rate of recurrence in the TS group was 6/55 (10.9%), and it was 6/81 (7.4%) in the TC group. No significant difference was detected in the rate of recurrence between the two groups (OR 1.02, 95% CI 0.22–4.80; p = 0.98; Fig. 5). The I^2^ statistic of 26% indicated no significant heterogeneity among included studies.

#### Meta-analyses of postoperative complications

**Figure 6:**
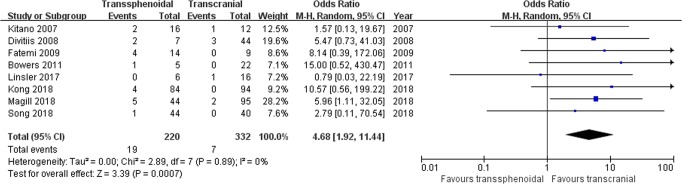
Forest plot of all studies with their respective OR and 95% CI, the number of events (cerebrospinal fluid leakage), and overall OR.

Cerebrospinal fluid leakage: Eight studies comprising 552 patients were included for the random-effects meta-analysis^15,21,22,28–32^. The rate of CSF leakage in the TS group was 19/220 (8.6%), and it was 7/332 (2.1%) in the TC group. The meta-analysis of pooled data showed a significantly higher risk from the TS approach with respect to the rate of CSF leakage (OR 4.68, 95% CI 1.92–11.44; p = 0.0007; Fig. 6). The I^2^ statistic of 0% indicated no significant heterogeneity among included studies.

**Figure 7:**
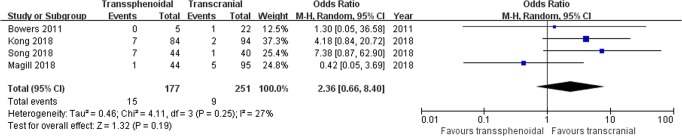
Forest plot of all studies with their respective OR and 95% CI, the number of events (infection), and overall OR.

Infection: Four studies comprising 428 patients were included for the random-effects meta-analysis^22,28,30,31^. The rate of infection in the TS group was 15/177 (8.5%), and it was 9/251 (3.6%) in the TC group. The meta-analysis of pooled data showed a higher risk from the TS approach in the rate of infection (OR 2.36, 95% CI 0.66–8.40; p = 0.19; Fig. 7), but the difference was not statistically significant. The I^2^ statistic of 27% indicated no significant heterogeneity among included studies.

**Figure 8:**
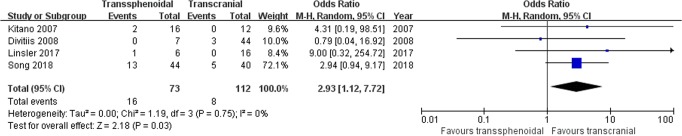
Forest plot of all studies with their respective OR and 95% CI, the number of events (dysosmia), and overall OR.

Dysosmia: Four studies comprising 185 patients were included for the random-effects meta-analysis^15,29,31,32^. The rate of dysosmia in the TS group was 16/73 (21.9%), and it was 8/112 (7.1%) in the TC group. The meta-analysis of pooled data showed a significantly higher risk from the TS approach in the rate of hyposmia (OR 2.93, 95% CI 1.12–7.72; p = 0.03; Fig. 8). The I^2^ statistic of 0% indicated no significant heterogeneity among included studies.

**Figure 9:**
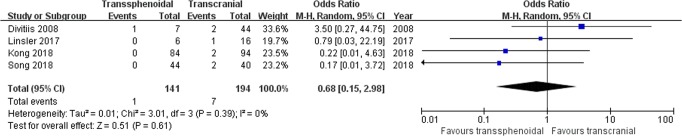
Forest plot of all studies with their respective OR and 95% CI, the number of events (intracranial hemorrhage), and overall OR.

Intracranial hemorrhage: Four studies comprising 335 patients were included for the random-effects meta-analysis^15,28,29,31^. The rate of intracranial hemorrhage in the TS group was 1/141 (0.7%), and it was 7/194 (3.6%) in the TC group. No significant difference was detected in the rate of intracranial hemorrhage between the two groups (OR 0.68, 95% CI 0.15–2.98; p = 0.61; Fig. 9). The I^2^ statistic of 0% indicated no significant heterogeneity among included studies.

**Figure 10:**
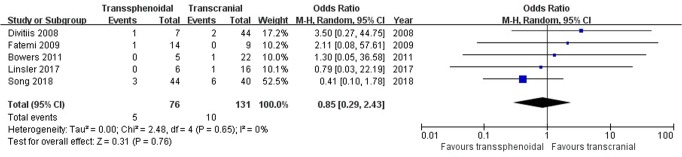
Forest plot of all studies with their respective OR and 95% CI, the number of events (endocrine disorders), and overall OR.

Endocrine disorders: Five studies comprising 207 patients were included for the random-effects meta-analysis^15,21,22,29,31^. The rate of endocrine disorders in the TS group was 5/76 (6.6%), and it was 10/131 (7.6%) in the TC group. No significant difference was detected in the rate of endocrine disorders between the two groups (OR 0.85, 95% CI 0.29–2.43; p = 0.76; Fig. 10). The I^2^ statistic of 0% indicated no significant heterogeneity among included studies.

Publication bias and quality assessment: The funnel plots for clinical outcomes and postoperative complications were overall symmetrical, suggesting no obvious publication bias (see Supplementary Fig. S1–8). The NOS sore of included studies ranged from 7 to 9.

## Discussion

Given the proximity to vital neurovascular structures, resection of TSMs remains a substantial challenge despite the remarkable advances in surgical techniques and approaches for skull base tumors.

Previous studies provided various levels of evidence about the approach selection in TSMs. Clark *et al*.^16^ performed a meta-analysis that included 6 studies involving 49 patients in the TS group and 11 studies involving 412 patients in the TC group demonstrating the association between the TS approach and higher rates of CSF leakage and visual improvement as well as no difference in extent of resection and morbidity. Graffeo *et al*.^20^ and Muskens *et al*.^19^ conducted meta-analyses involving more case series yielding similar results. In addition, Graffeo *et al*.^20^ revealed a higher risk of recurrence associated with the TS approach. Muskens *et al*.^19^ revealed that TS approach was associated with higher rates of intraoperative arterial injury. The reliability of these reviews was diminished because of including non-comparative studies.

In the past, with respect to the TS surgery, there were some apparent drawbacks, such as unclear tumor exposure, limited operation experiences under an endoscopic view, and lack of reliable sellar reconstruction. These operative limitations gave rise to the debate regarding the application of the TS approach in resection of TSMs. However, with advances in related techniques, neurosurgeons performed the TS approach surgery in selected patients with TSMs, achieving a remarkable rate of gross resection with an acceptable rate of CSF leakage^12,33,34^. Thus, many centers started to alternatively use the TS approach in selected patients. In this study, we enrolled data from comparative studies. The purpose of this meta-analysis was to comprehensively compare the clinical outcomes and postoperative complications of removing TSMs between the TS and TC approaches.

Traditionally, tumor resection, tumor recurrence, visual improvement, and CSF leakage were the most focused surgical results. In the present study, we further evaluated the impact of approach selection on common postoperative complications, which drew little attentions previously but were closely related to the recovery course and quality of life.

The extent of tumor resection is the strongest independent prognostic factor for meningioma^35^. With respect to the rate of GTR, our meta-analysis did not show a significant difference between the TS and TC groups. The result was consistent to previous studies. However, in the early years of the endoscopic surgery, the selection bias existed resulting in the inclusion of patients with smaller tumor size and less complicated anatomical relationships into the TS group. Tumor size, lateral extension, and neurovascular encasement contribute to the extent of tumor resection rather than the surgical approach alone. In three studies^22,28,31,32^, baseline tumor size was balanced between groups, mostly being less than 30 mm in diameter. Optic canal invasion was compared at baseline in four studies^15,28,30,31^, of which three studies showed no significant difference between groups^15,30,31^. The TS surgery is more suitable for tumors with small size (<30 mm) and limited extension whereas the complex anatomy is the indication for the TC surgery for sure. In our perspective, along with constant improvement of the endoscopic instruments and skills, endoscopic visualization can detect lateral extension easily and offer high-definition intraoperative images, allowing for more delicate surgical manipulation for complex TSMs in the future.

The most common initial presentation of TSMs is visual disturbance due to tumor extension to the optic canal^36^. Therefore, the visual change is considered as a major surgical indication for TSMs, and the restoration and preservation of visual function are the primary goals of surgical treatment. Visual improvement is defined as either or both of improved visual acuity and visual field in our study. Tumor features including size, extension and duration of preoperative visual dysfunction are critical predictive factors for visual recovery. Tumor size and extension were balanced in almost half of included studies as we mentioned above. Unfortunately, included studies didn’t bring much attention to detailed neuroimaging characteristics and ophthalmologic examination results. Therefore, we proposed that the ophthalmologist should be a member of the research team for TSMs. Considering the growth pattern of TSMs, prompt and complete optical canal decompression is associated with better visual outcomes. The results of visual outcomes in different studies are more favorable in the TS approach. Two key explanations are given to elucidate the phenomenon as following: (1) minimized manipulation of the optic nerves and optic chiasm; (2) well protection of blood supply to optic nerves. In contrast, in the TC approach, brain retraction and manipulation of neurovascular tissues are indispensable for tumor exposure and are likely to cause iatrogenic injuries to the optic nerves. Meanwhile, Julien *et al*.^37^ also suggested that contralateral transcranial approach could allow preservation of vascular supply and less mobilization of the compromised optic nerve. Apart from the approach selection, tumor size must be taken into consideration because tumor size is the most important factor affecting preoperative visual function. In the present study, six studies were included for the analysis of visual outcomes in each group. Among three over six included studies^28,31,32^, tumor size was compared at baseline and showed no significant difference between groups. When including the three high-level studies into a meta-analysis, the results were also in favor of the TS approach. Thus, it is believed that visual recovery is more favored in the TS group for patients with small tumor size (<30 mm) and limited extension.

In our study, recurrence is defined as tumor regrowth after complete resection. Pathologically, TSMs are commonly WHO I meningiomas classified as benign tumors. Therefore, considering similar rate of GTR between groups, recurrence is the most reliable indicator to evaluate the long-term clinical outcome. Theoretically, the TS approach is more convenient to remove affected bone and dura, achieving Simpson I resection and decreasing the risk of recurrence. On one hand, for a relatively new technique for tumor resection, the mean follow-up period in prior studies is not long enough for each individual to completely estimate its effect on tumor recurrence. On the other hand, the learning curve may undermine the real long-term outcome of the TS approach for TSM resection in experienced hands. The present data showed no significant difference between groups. From our view, the impact of approach selection on TSM recurrence will be better clarified with the data accumulation regarding longer follow-up results and more experienced TS resection of TSMs.

Though the TS approach has many advantages, its drawbacks are non-negligible, such as the difficulty in reconstructing cranial base dura and bone defects. CSF leakage is a significant complication of adopting the TS approach though it is commonly not associated with additional mortality unless surgical repairing is required. In our meta-analysis, the TS approach is associated with a significant higher rate of CSF leakage. The finding is consistent to previous studies. However, there is an obvious decreasing risk of CSF leakage as the neurosurgeons become more experienced in TS approach and skull base closure techniques.

Postoperative infection is generally associated with many factors such as blood loss, operation time, CSF leakage and surgical incision. The TS approach is associated with significant shorter operation time and less amount of bleeding compared with the TC approach. The higher risk of CSF leak and intranasal bacteria may account for higher rate of infection in the TS group though there is no significant difference between groups. It is commonly viewed that the most important factor of infection is CSF leakage in patients receiving a TS surgery. As the technology of skull base reconstruction improves, the rate of CSF leakage-associated infection in the TS group is bound to be low in the future.

Dysosmia is a notable complication because diminished olfaction after surgery is associated with a poor quality of life. Olfaction dysfunction is attributed to damage of olfactory nerve in the TC approach. In the TS approach, the superior trajectory frequently skirts the ethmoid sinuses and damages olfaction. In addition, excessive abrasion of the nasal mucosa also accounts for olfactory disturbance.

The risks of intracranial hemorrhage and endocrine disorders are not affected by the approach selection according to our result of data synthesis. The rate of intracranial hemorrhage is higher in the TC group with no significant statistical difference. Intracranial hemorrhage, which is extremely rare in a TS procedure, is generally considered as a craniotomy-specific complication. As the craniotomy skill improves, the risk of intracranial hemorrhage has been lowered to an acceptable level in clinical practice^38^. Endocrine disorders after resection of TSMs mainly consist of diabetes insipidus and hypopituitarism. The symptoms were transient or persistent requiring replacement therapy.

### Study Limitations

The present study had several limitations. First, only 8 studies were included in the meta-analysis. More studies are warranted to compare the surgical results of different approaches. Second, the pooled data were all from retrospective studies with inherent selection and treatment bias. The evaluation of clinical symptoms, selection of patients, tumor size, and definition of gross total resection were methodologically variable among studies. Tumor location and extension, which were important for further stratification of results, were not well documented or balanced at baseline in all included studies. Thus, it is difficult to make a categorical conclusion for surgical route selection. Third, the time span of patient inclusion was long, ranging from 1983 to 2016, during which both the TS and TC techniques developed rapidly. In addition, as a novel technique, the learning curve existed in the early era of TS surgery. The above drawbacks may limit the application of our conclusions in clinical practice. Despite these limitations diminishing the comparability of the two surgical routes, to date, this meta-analysis provides the most convincing evidence in selection of surgical approach for TSMs. More prospective researches are needed to further determine the optimal use of the two approaches in specific patients.

## Conclusions

Our findings recommend that the TS approach with minimal invasive features serves as a safe and effective alternative to the TC approach for TSM treatment in selected cases with small tumor size and limited optic canal invasion. The major risk of TS approach is CSF leakage, which is deemed to decrease in parallel with ongoing improvements of surgeon experiences and skull base reconstruction techniques.

## Supplementary information

Supplementary figures
