# Supplementary material for: Transsphenoidal versus Transcranial Approach for Treatment of Tuberculum Sellae Meningiomas: A Systematic Review and Meta-analysis of Comparative Studies
Source: Sci Rep. 2019 Mar 19;9:4882. doi: 10.1038/s41598-019-41292-0 (PMC6424979; doi:10.1038/s41598-019-41292-0)

Transsphenoidal versus Transcranial Approach for Treatment of Tuberculum Sellae Meningiomas: A Systematic Review and Meta-analysis of Comparative Studies.

Chengxian Yang, Yanghua Fan, Zhiwei Shen, Renzhi Wang & Xinjie Bao

Department of Neurosurgery, China Pituitary Disease Registry Center, Peking Union Medical College Hospital, Peking Union Medical College & Chinese Academy of Medical Sciences, Beijing, 100730, China

Correspondence and requests for materials should be addressed to X.B. (email: baoxinjie1@pumch.cn)

Supplementary Figure S1. Funnel plot showing symmetrical distribution of studies, indicating absence of publication bias (visual outcome).

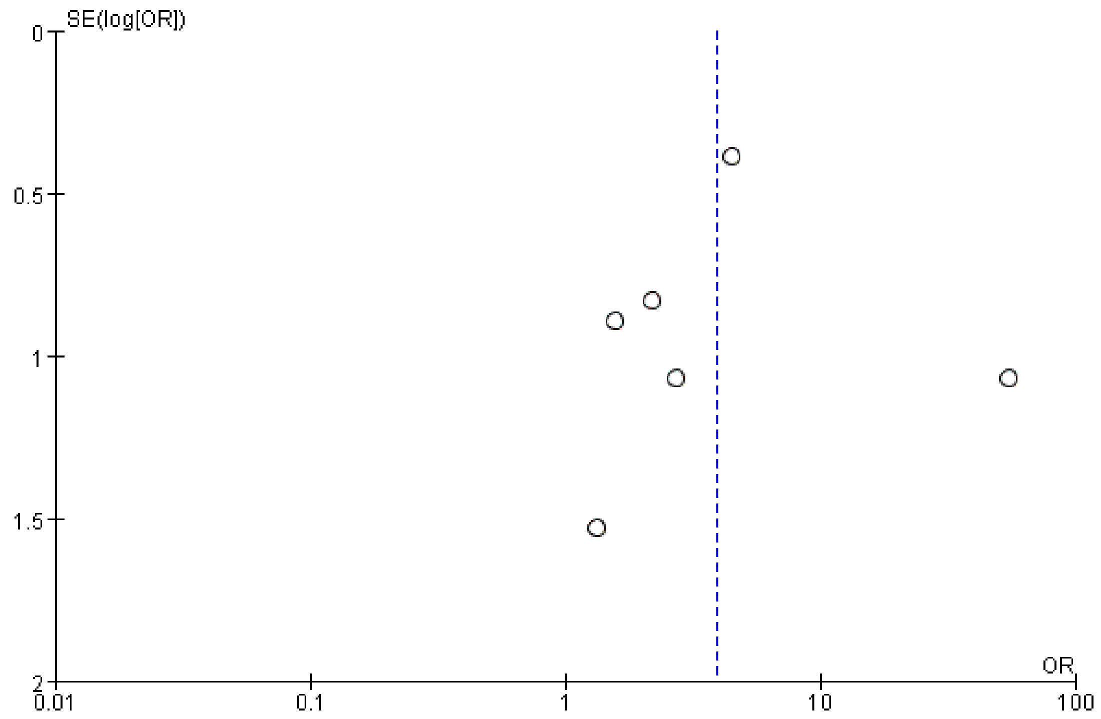

Supplementary Figure S2. Funnel plot showing symmetrical distribution of studies, indicating absence of publication bias (tumor resection).

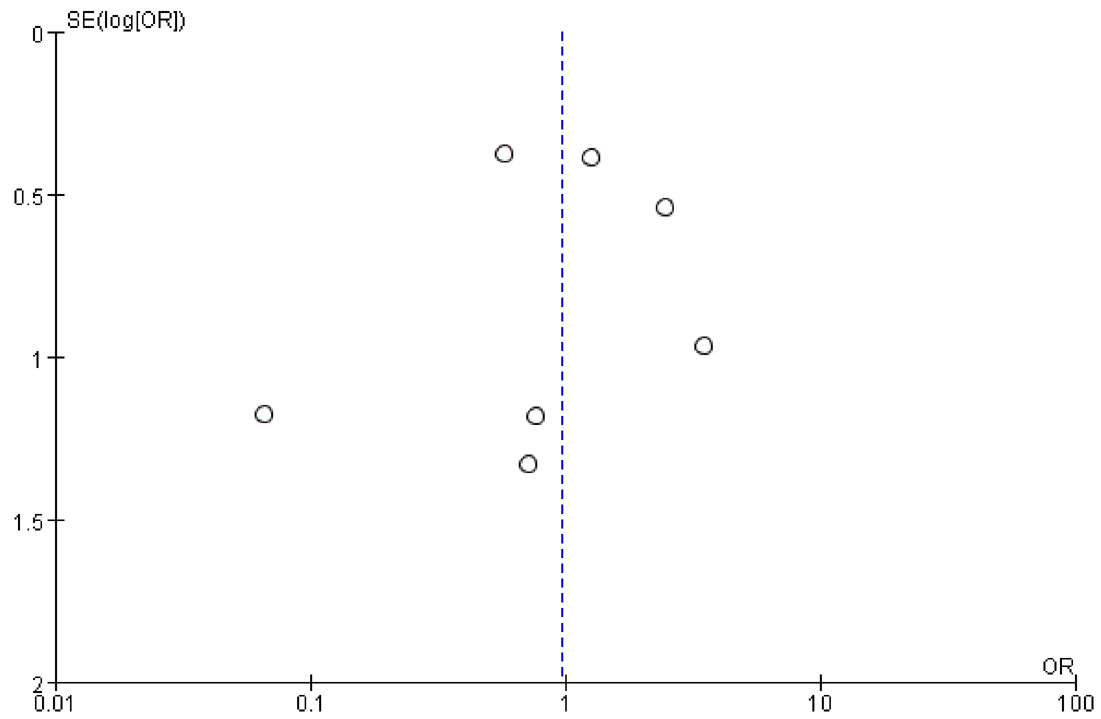

Supplementary Figure S3. Funnel plot showing symmetrical distribution of studies, indicating absence of publication bias (recurrence).

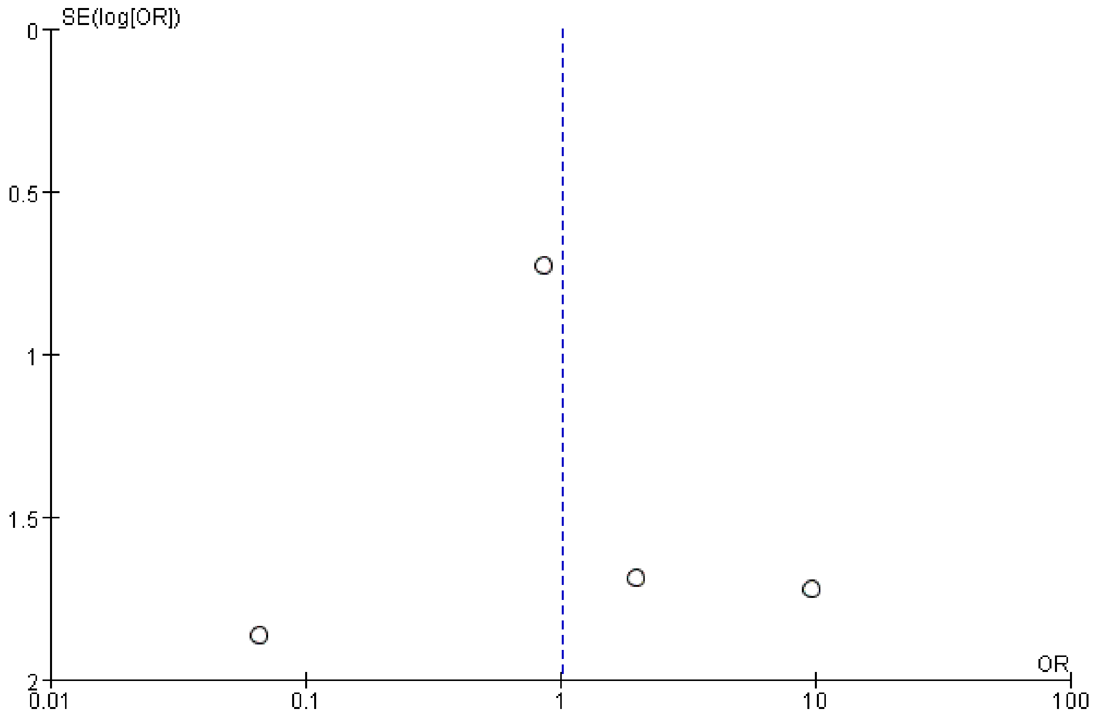

Supplementary Figure S4. Funnel plot showing symmetrical distribution of studies, indicating absence of publication bias (cerebrospinal fluid leakage).

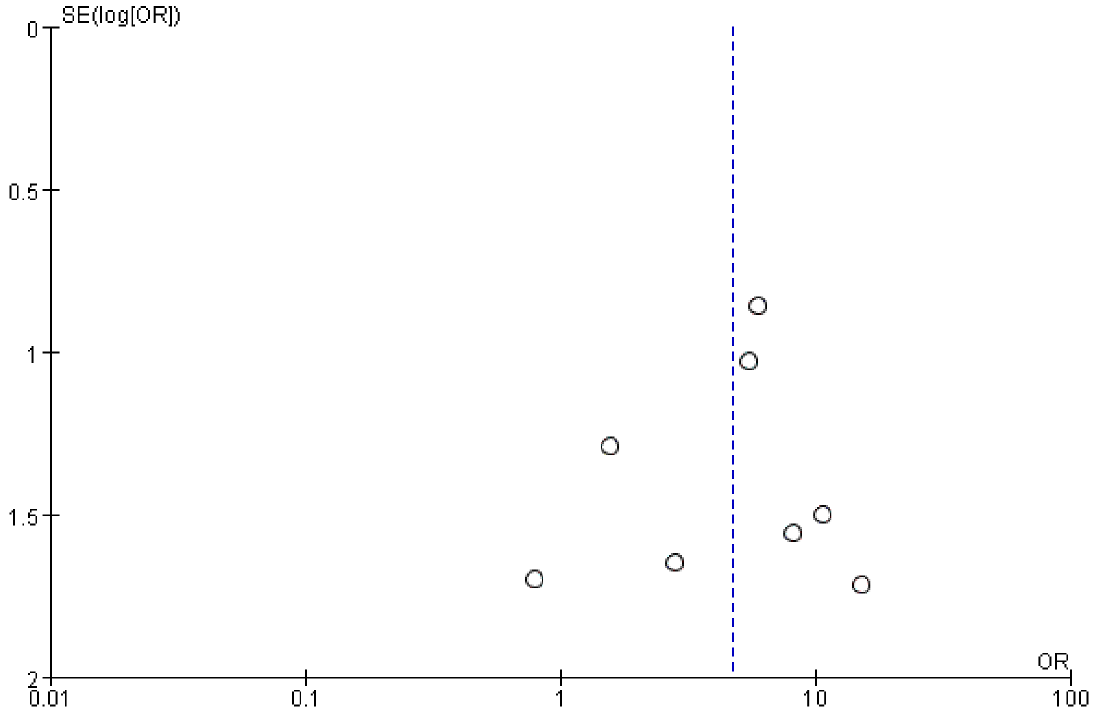

Supplementary Figure S5. Funnel plot showing symmetrical distribution of studies, indicating absence of publication bias (infection).

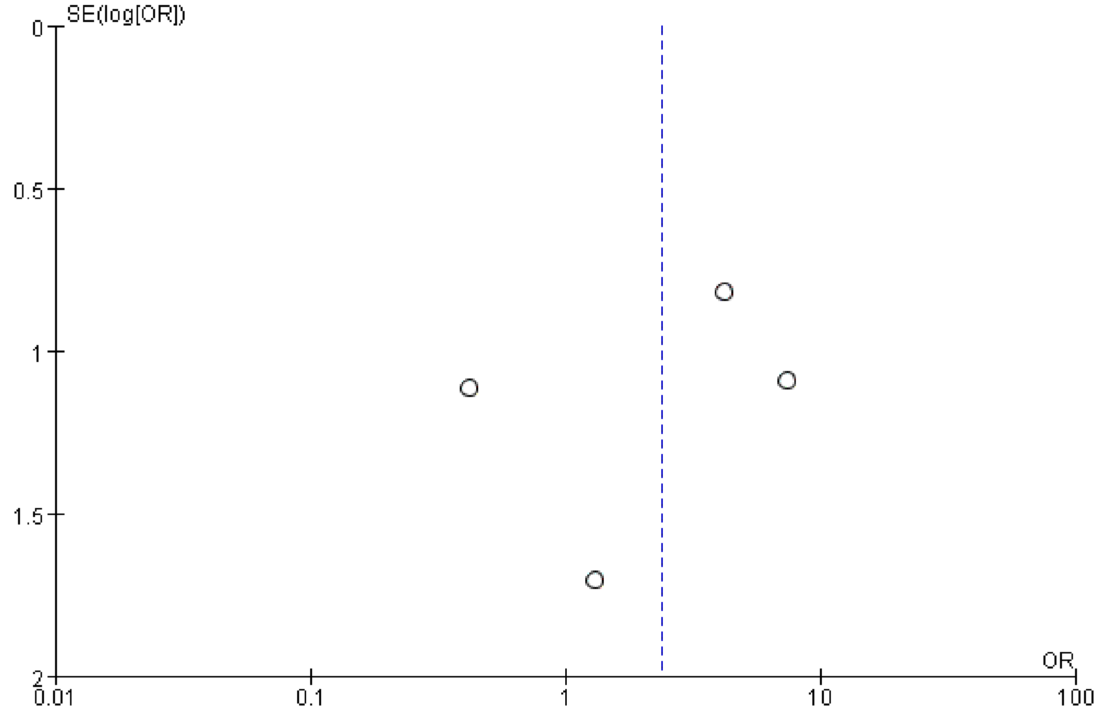

Supplementary Figure S6. Funnel plot showing symmetrical distribution of studies, indicating absence of publication bias (dysosmia).

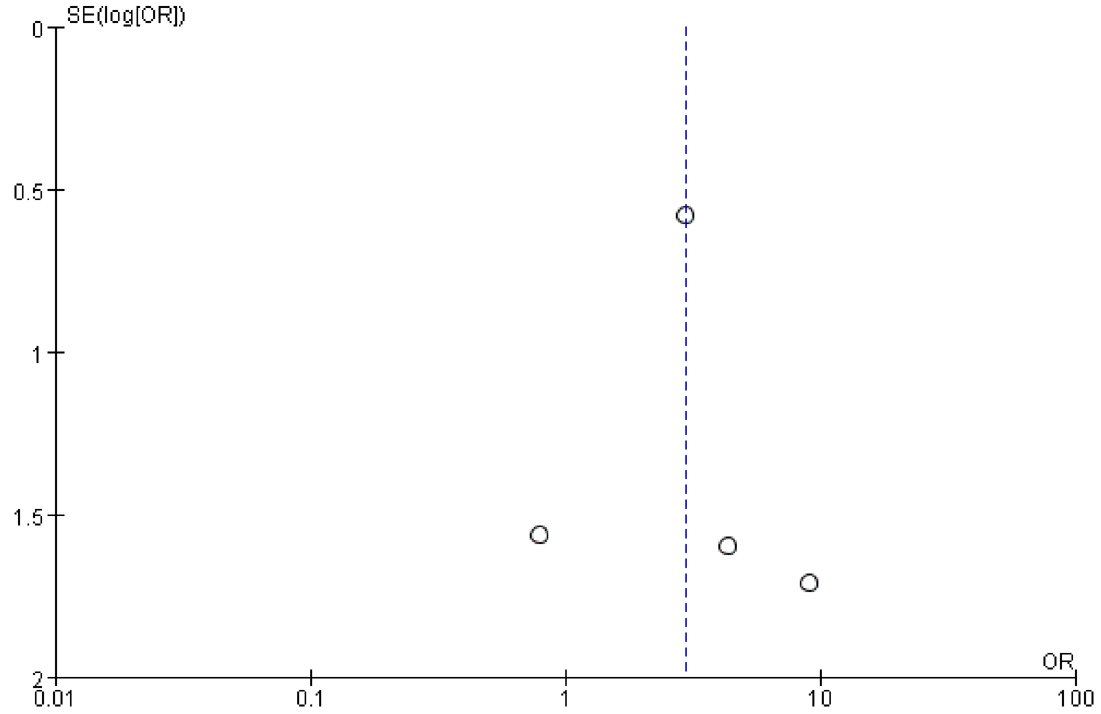

Supplementary Figure S7. Funnel plot showing symmetrical distribution of studies, indicating absence of publication bias (intracranial hemorrhage).

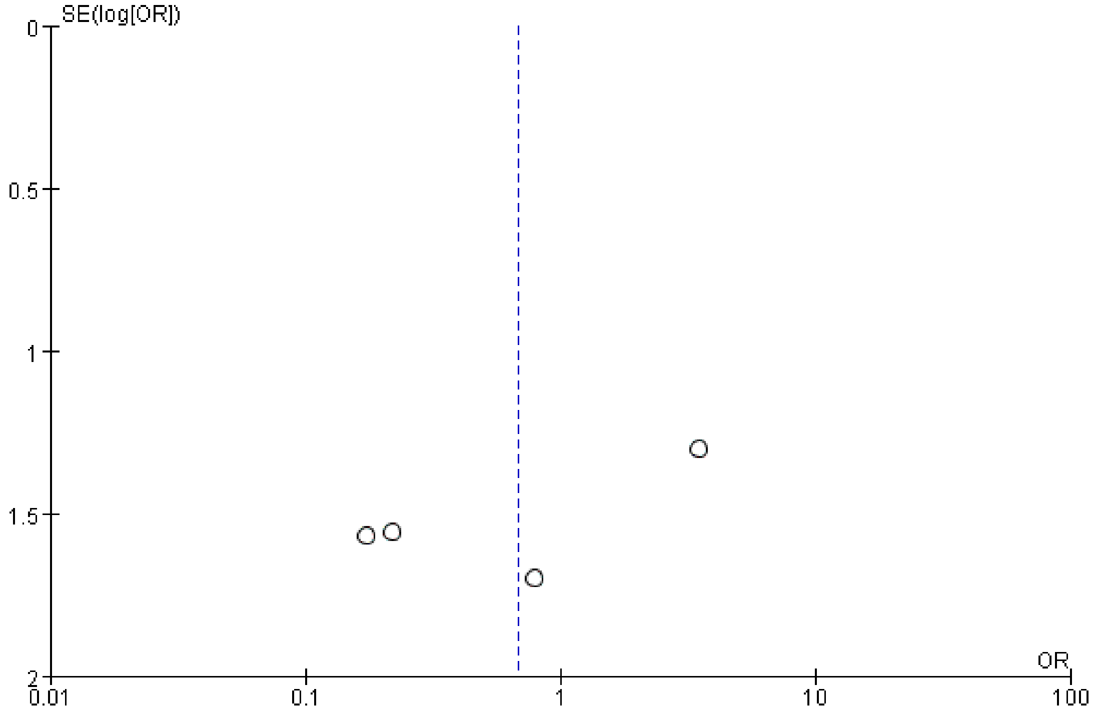

Supplementary Figure S8. Funnel plot showing symmetrical distribution of studies, indicating absence of publication bias (endocrine disorders).

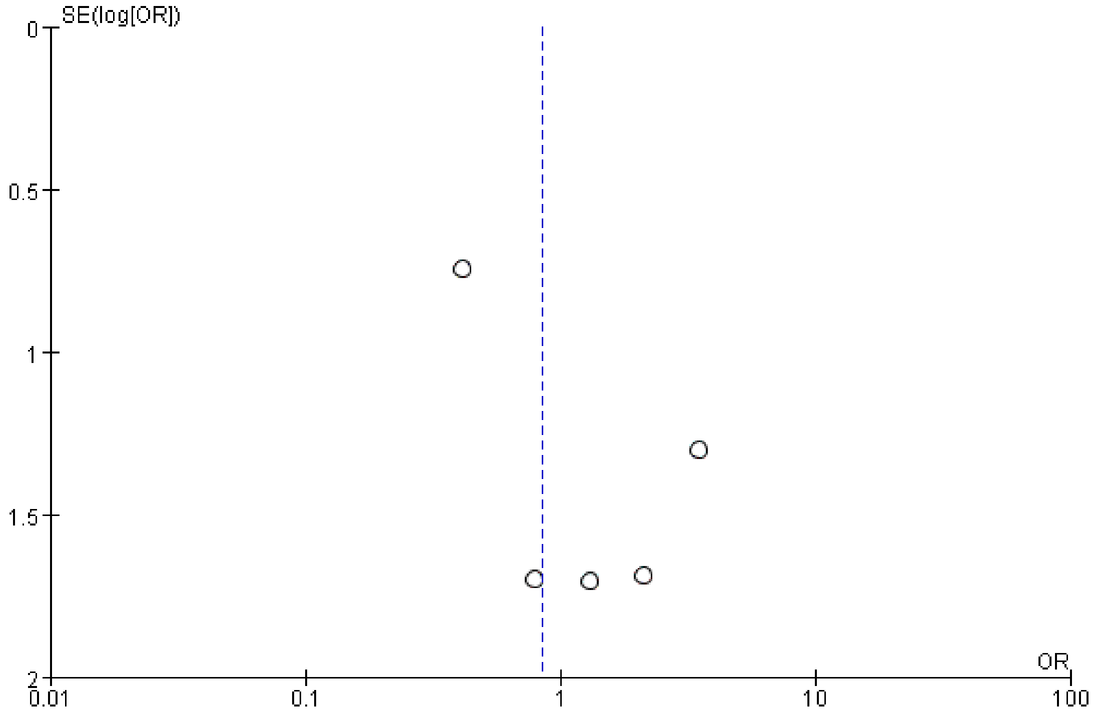

Supplement: Supplementary file 1 — Supplementary figures [file 41598_2019_41292_MOESM1_ESM.pdf]
